# Type 2 Brugada Electrocardiogram Pattern Due to Supra-Therapeutic Phenytoin Level

**DOI:** 10.7759/cureus.14381

**Published:** 2021-04-09

**Authors:** Haris Iftikhar, Khalid Bashir

**Affiliations:** 1 Emergency Medicine, Hamad Medical Corporation, Doha, QAT

**Keywords:** ecg, brugada syndrome, brugada ecg pattern, brugada phenocopy, phenytoin, phenytoin overdose

## Abstract

Brugada syndrome (BS) is a hereditary cardiac disease leading to sudden cardiac death. It does not display any structural cardiac abnormalities. It was first described in 1992, as the syndrome of ‘right bundle branch block, persistent ST segment elevation, and sudden death.’ Brugada phenocopy (BP) is a relatively new term used to describe electrocardiogram (ECG) patterns that resemble BS but are due to other reversible causes such as electrolyte abnormalities, fever, cocaine or alcohol intoxication, and side effect of certain medications such as sodium channel blockers, beta blockers, antidepressants, alpha adrenergic blockers, etc. Earlier studies have shown that patients taking sodium channel blocking antiepileptic drugs (AEDs) especially phenytoin can have Brugada type 1 like ECG pattern. Previously, type 2 ECG pattern secondary to supra-therapeutic phenytoin level has not been described. We describe a case with type 2 Brugada ECG pattern due to supra-therapeutic phenytoin level; the ECG pattern completely resolved following lowering the phenytoin to a therapeutic level. These patients need special considerations in ED management, disposition, and follow-up.

## Introduction

Brugada syndrome (BS) is a congenital cardiac condition that can lead to the sudden development of pulseless ventricular tachycardia (VT) and ventricular fibrillation (VF) leading to sudden cardiac death. BS was first described in 1992 [1]. Patients with BS do not have any structural heart disease but present with electrocardiogram (ECG) abnormalities typically showing a right bundle branch block and ST elevations in precordial leads one to three, hence it is believed to have electrical pathology [2-3]. Recently BS has been linked to mutation of cardiac sodium channel gene (SCN5A) further supporting the hypothesis that it is mainly an electrical disease [4]. The ECG pattern in BS may be dynamic and only apparent in the precordial leads which is the hallmark of this condition [5-6]. In 2012, the revised criteria for the diagnoses of BS was brought forward and consist of two types only, type 1 (coved pattern) and type 2, which is a combination of previously defined types 2 and 3 (saddleback pattern) [2]. 

A new term Brugada phenocopy (BP) has been coined to describe several other conditions causing an ECG appearance similar to BS but genetically dissimilar to BS. Most of these conditions are reversible such as adrenal insufficiency, hypokalemia, myocardial ischemia, and antiepileptic drugs (AEDs). Once the underlying condition is treated the ECG pattern returns back to normal. This is in contrast to true congenital BS where the ECG pattern is permanent [7-11].

In the literature, Class 1B antiarrhythmic drugs like phenytoin, which is also an antiepileptic drug, has been reported to induce type 1 Brugada pattern on ECG when this drug is present in supra-therapeutic levels in patients with epilepsy [12-13]. A study demonstrated that BS type ECG changes were observed in 12.5% of patients with seizure disorder taking AEDs [14]. Sodium channel blocking medications used in epilepsy may modify ventricular depolarization/repolarization leading to BS type ECG changes [5].

We report a case of type 2 Brugada ECG pattern in a patient with supra-therapeutic phenytoin level. Our case can also be considered as a case of BP due to supra-therapeutic dose of phenytoin, although, it does not meet the strict type 2 criteria proposed by Baranchuk et al. [10].

## Case presentation

A 29-year-old patient was brought to ED by his roommates in an ambulance when they witnessed an episode of generalized tonic-clonic seizure leading to loss of consciousness and postictal confusion. He denied tongue biting, urinary or bowel incontinence, or any trauma. There was no aura, chest pain, palpitations, or shortness of breath before or after the episode. His significant past medical history includes previous episodes of seizures, irritable bowel syndrome, and nonspecific dizziness. There was no family history of cardiovascular diseases including sudden cardiac death. He works as a laborer.

Following vital signs were observed in ED. Temperature of 37.2°C, respiratory rate 18 breaths/minute, blood pressure 136/74 mmHg, and SPO2 99% on room air. He had no signs of head trauma and had moist mucous membranes with no tongue bite. His cardiovascular and respiratory exam was unremarkable. His abdomen was soft and nontender with no organomegaly. His neurological exam showed normal Glasgow Coma Scale (GCS) with intact cranial nerves. There was no nystagmus and his motor exam was normal with no neurological deficit. He has no cerebellar signs and his gait was normal. His investigations showed normal complete blood count, C-reactive protein, renal function panel, calcium, magnesium, phosphorus, lactic acid, liver function test, thyroid-stimulating hormone, and urine dipstick. His CT head was also unremarkable. 

**Figure 1:**
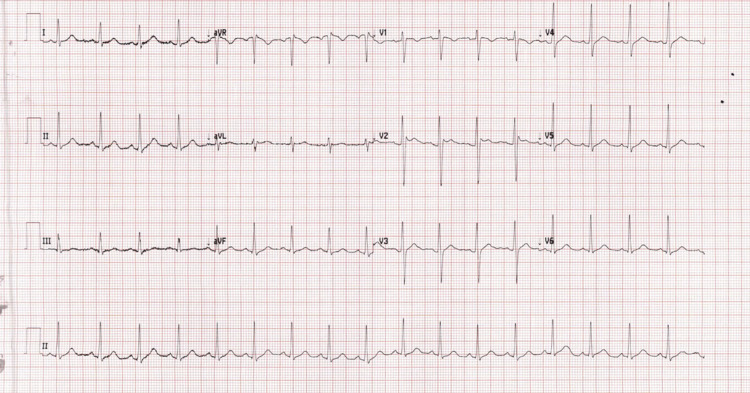
12-Lead ECG showing type 2 Brugada pattern. ECG, electrocardiogram

**Figure 2:**
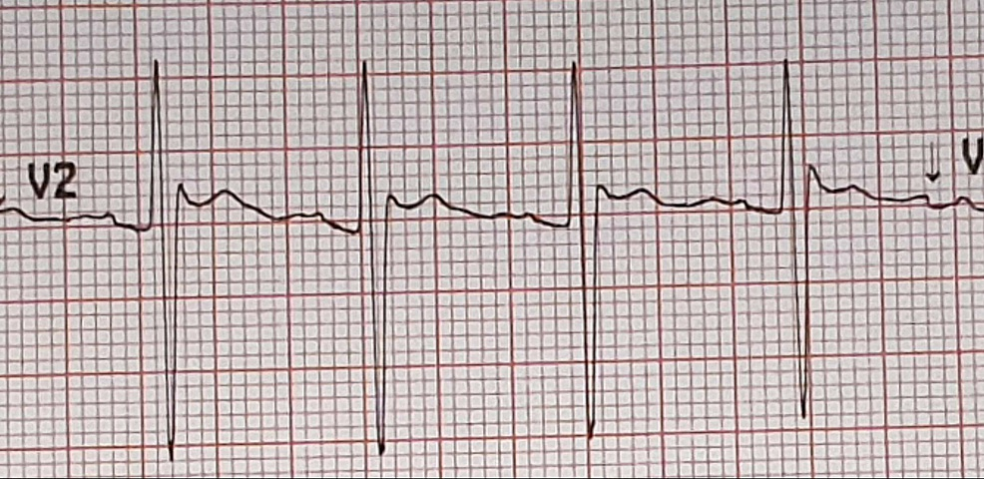
Lead V2 showing high take-off of ST segment elevation with a J wave amplitude of more than 2 mm and gradually descending ST segment elevation of more than 1 mm above baseline.

Based on the history and examination findings, the diagnosis of epilepsy was made. He was loaded with phenytoin according to local protocols, considering medication affordability issues. He was given 1500 mg IV in 500 mL normal saline over one hour. His weight was 80 kg. He received 18.75 mg/kg of an IV loading dose of phenytoin. About 20 min after starting the phenytoin infusion, the patient developed severe dizziness. It was so severe that the patient was unable to sit or stand. There was no nausea, vomiting confusion, or slurred speech associated with dizziness. He had nystagmus on his eye exam. His pupils were equal, round, and reactive to light. His GCS was 15. His cranial nerve exam was normal. He had limb ataxia, but his gait was not assessed. Otherwise, he had no motor or sensory deficits. His random blood sugar (RBS) was 5.6 mmol/L. His ECG was done that showed high take-off of ST segment elevation in V2 with J wave amplitude of more than 2 mm and gradually descending ST segment elevation of more than 1 mm above baseline meeting criteria for type 2 Brugada pattern (Figures 1-2) [9]. His phenytoin level was done 8 h from the loading dose which was 127.6 umol/L (therapeutic range: 40-79 umol/L). A toxicology consult was made and he was admitted to the inpatient observation unit.

Outcome and follow-up

**Figure 3:**
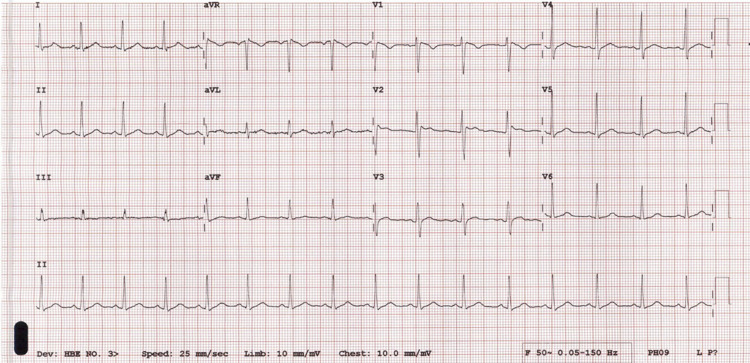
Repeat ECG with phenytoin level of 101.3 umol/L. ECG, electrocardiogram

**Figure 4:**
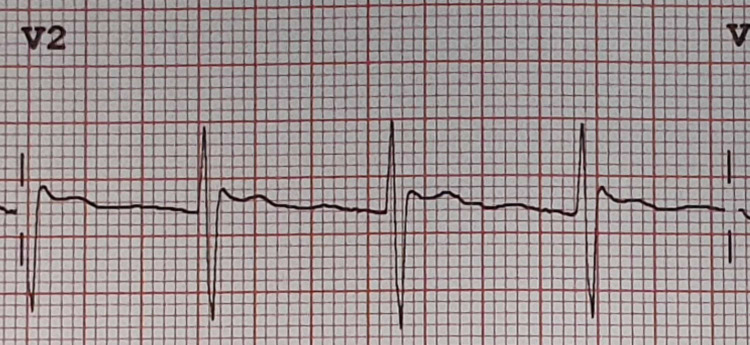
Lead V2 showed a decrease of J wave amplitude and ST segment elevation as compared to the first ECG. ECG, electrocardiogram

**Figure 5:**
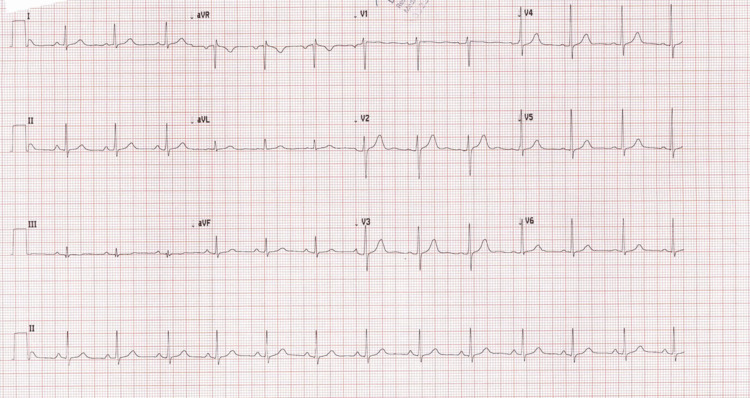
Normal ECG with a therapeutic level of phenytoin. ECG, electrocardiogram

The patient remained stable in the observation unit. Phenytoin level was repeated in the observation unit and the level was 101.3 umol/L 21 h after the loading dose. He still felt dizzy with nystagmus and gait ataxia. The ECG was repeated. The repeated ECG showed a type 2 Brugada pattern with a slight decrease of J wave amplitude and ST segment elevation as compared to the first ECG (Figures 3-4). Further phenytoin level was done at 46 h from loading dose which showed a level of 65.2 umol/L (therapeutic range: 40-79 umol/L). His symptoms were completely resolved at this time. His ECG findings returned to normal (Figure 5). The case was discussed with neurology department. He was discharged home with a 300 mg phenytoin tablet daily at bedtime. He was given an outpatient referral to the neurology department. 

## Discussion

After the literature search, we believe that this is the first case of type 2 Brugada pattern as a result of supra-therapeutic phenytoin level. There are two previous case reports of type 1 Brugada pattern due to supra-therapeutic phenytoin level [12-13]. Type 2 ECG pattern has not been reported probably due to the reason that type 2 or previously classified type 2 and 3 patterns are less significant in causing BS and related arrhythmias [2]. Patients with type 2 ECG patterns are important in the ED in terms of their management, disposition, and follow-up plan. These patients need a thorough history to exclude palpitations, arrhythmia, syncope, or family history of sudden cardiac death. They need a repeat ECG on discharge to confirm if the ECG change is actually due to a reversible cause. If history is concerning for BS, they will need cardiology follow-up for testing for true BS [10]. 

For investigating the high-risk patients with suspected BS with a normal ECG, a drug challenge can be introduced. Class IA arrhythmogenic drugs such as flecainide, procainamide, disopyramide, and propafenone have been used to unmask BS in high-risk patients [3, 5]. These drugs may induce or exaggerate the appearance of type 1 and type 2 patterns. There are no reliable pharmacological interventions available for the treatment or prevention of BS. The only effective treatment for BS is the insertion of an automated implantable cardiac defibrillator (ICD) which efficiently treats VT and VF and prevents sudden cardiac death [2]. Radiofrequency catheter ablation has also been recommended as an alternative treatment [6].

One small retrospective observational study done by Ishizue et al. on 120 patients with epilepsy who regularly take therapeutic doses of AEDs and had ECGs recorded during these therapies found 15 patients exhibited Brugada-type ST elevation. Previous studies have reported that the prevalence of Brugada-type ST elevation was 0.10%-0.15% in the general population [14], however, in this research, Brugada-type ST elevation was noted in 15/120 (12.5%) patients. Phenytoin was used in a higher percentage of patients with Brugada-type ST elevation than in those without it. Polytherapy with sodium channel-blocking AEDs was more frequently observed in patients with Brugada-type ST elevation. The study concluded that Brugada-type ST elevation was noted in higher rates in patients with epilepsy and those taking AEDs than those in the general population [14].

There is a need for more data on ECG changes in epileptic patients taking AEDs. Emergency physicians need to be aware of the findings that Brugada-type ECG pattern can be observed in patients taking AEDs, particularly with supra-therapeutic levels. Type 2 Brugada ECG pattern although less significant than type 1 can be observed in patients with phenytoin overdose.

## Conclusions

Type 2 Brugada pattern may develop in patients presenting with symptoms secondary to a supra-therapeutic dose of phenytoin. It is important for emergency physicians to understand that the type 2 Brugada pattern can be reversible and is less significant than type 1, so that appropriate evaluation and treatment is offered.
